# Reliability and agreement study of three-dimensional measurement for femoral head displacement indicators after femoral neck fractures

**DOI:** 10.1038/s41598-026-41210-1

**Published:** 2026-02-27

**Authors:** Shuangshuang Cui, Jingbo Yu, Likun Zhao, Shujun Yu, Jianxiong Ma, Xinlong Ma

**Affiliations:** 1 Tianjin Hospital, Tianjin University, Tianjin, 300211 People’s Republic of China; 2 Orthopaedics Institute of Tianjin, Tianjin Hospital, Tianjin, 300211 People’s Republic of China; 3 Tianjin Key Laboratory of Orthopaedic Biomechanics and Medical Engineering, Tianjin, 300211, People’s Republic of China; 4 Department of Orthopaedics, Tianjin Hospital, Tianjin, 300211 People’s Republic of China

**Keywords:** 3D reconstruction, Displacement of femoral head, Inter-observer, Reliability, Agreement, Anatomy, Diseases, Health care, Medical research

## Abstract

The outcomes of femoral neck fractures (FNFs) are influenced by the displacements of femoral head preoperatively and the reduction quality of surgery. CT-based three-dimensional (3D) reconstruction and measurement techniques have been widely employed in numerous studies to evaluate femoral head displacements both preoperatively and postoperatively. This study aims to measure specific indicators of displacement and residual displacement of femoral head after FNFs, and to assess the reliability and agreement of the measurement methods. Pre- and post-operative CT data of FNF patients treated with cannulated screws (2015–2020) were measured and analyzed. Bilateral proximal femurs were reconstructed using Mimics 22.0. Key measurements included: displacement of fovea of femoral head (d1), displacement of center of femoral head (d2), and 3D angle (α). The measurements were carried out by three experienced observers independently. Intraclass Correlation Coefficient (ICC) and Concordance Correlation Coefficient (CCC) were used to evaluate reliability and agreement. The standard error of measurement (SEM), Bland-Altman plots and 95% limits of agreement (LoA) were used to evaluate agreement. This study analyzed 200 CT scans from 100 patients (63.5 ± 4.9 years; 56 F/44 M). All measurement parameters (d1, d2, α) showed significant changes (*P* < 0.001) after fixation. Inter-observer reliability and agreement were great (ICCs: 0.894–0.982; CCCs: 0.737–0.946) for preoperative indicators and good-to-moderate (ICCs: 0.605–0.846; CCCs: 0.336–0.644) for postoperative indicators. Bland-Altman plots revealed acceptable agreement for all comparisons between two observers pre- and postoperatively with ≥ 92% data points falling within 95% LoAs. The SEMs for most measurement indicators were low both preoperatively and postoperatively (range: 0.857–3.565), except for angles measured in the transverse and sagittal planes. This study confirms that CT-based 3D reconstruction is valid for preoperative femoral neck fracture assessment (ICC > 0.894). While postoperative measurements show moderate agreement and greater measurement error, particularly in the sagittal plane, it should be noted that ICC/CCC values can be influenced by variability among samples. In future research, it is essential to focus on the standardized registration of models across all three anatomical planes during 3D reconstruction to further enhance measurement accuracy and stability.

**Supplementary Information:**

The online version contains supplementary material available at 10.1038/s41598-026-41210-1.

## Introduction

Epidemiological studies have shown that femoral neck fractures (FNFs), which are anatomically classified as intracapsular hip fractures, account for approximately 50% of all hip fracture cases^1,2^. Hip fracture patients are predominantly frail elderly individuals with multiple comorbidities, whose overall prognosis, particularly mortality, is significantly influenced by preoperative and perioperative comorbidities. Studies have confirmed that specific preoperative cardiac risk factors^3^ and atrial fibrillation^4^ are important predictors of postoperative mortality in elderly patients. However, for the relatively younger and more active group of femoral neck fracture patients, the primary treatment goal is not only to reduce mortality but also to restore function through successful internal fixation and prevent long-term complications such as osteonecrosis of the femoral head (ONFH). Internal fixation is one of the primary treatments for FNFs, particularly in physiologically younger patients or those with good bone mass^1,5^.

Occurrence of ONFH following a FNF severely impacts patients’ quality of life^6^. Recent studies have shown that the incidence of ONFH following internal fixation of FNFs remains persistently high, reaching up to 14.5~30%^7,8^. The severity of FNFs and quality reduction of femoral head postoperatively are critical factors influencing clinical outcomes^9,10^. At present, standard methods for assessing fracture types (such as Garden classification and Pauwels classification) and reduction quality evaluation criteria (including the Garden alignment index and Lowell’s S-curve) are all still based on two-dimensional(2D) radiographs^11–14^. Even though a fracture is considered anatomically reduced on an X-ray, residual displacements can still exist in 3D space because of obstructions from overlapping structures and issues with positioning angles^11^. CT-based 3D reconstruction has shown that even Garden type I fractures, presumed non-displaced, exhibit significant displacement^15,16^.

With the widespread application of CT-based 3D reconstruction, it has become feasible to measure the morphology and variations of human anatomical structures. Tarek and colleagues^17,18^ employed a CT-based 3D “direct measurement” technique in their methodological studies to quantify the torsion angle of fracture ends in femoral neck models and to validate the technique’s feasibility and accuracy for malrotation assessment. Ma et al.^19^ achieved accurate, anatomically-defined femoral anteversion measurement through spherical fitting of the femoral head using 3D reconstruction. In 2010, Citak^20^ et al. demonstrated that CT-based 3D methods provided superior accuracy compared to conventional 2D techniques in the presence of positional variation. Subsequently, introduction of new automated algorithms (e.g., cylinder fitting) further reduced measurement variability by up to fivefold, significantly enhancing reliability and efficiency^21^. In 2012, Ma et al.^22^ firstly used CT-based 3D reconstruction and mirroring techniques to assess femoral head displacements after FNFs. Recently, an increasing number of studies are utilizing CT-based 3D reconstruction to assess femoral head displacements and residual displacements after reduction and fixation^23–25^. One previous study showed that the postoperative recovery and ONFH in patients with FNFs are influenced by the residual displacement of femoral head after surgery^26^. Wang et al.^11^ precisely measured the displacement of fovea, center and 3D angle of femoral head, and systematically evaluated the reduction quality following FNFs. The study conclusively demonstrated that inadequate reduction quality significantly increases the risk of ONFH. CT-based 3D reconstruction measurements demonstrate clinical potential for assessing both initial displacement following FNFs and residual displacement after reduction.

An increasing number of studies are employing CT-based 3D reconstruction to measure displacement in FNFs, highlighting the need for research into the reproducibility of these measurement techniques. The objective of this study was to evaluate the agreement and reliability among different observers in measuring femoral head displacement parameters using CT-based 3D reconstruction, thereby providing methodological validation for this measurement approach.

## Methods and population

### Study design and population

Pre- and post-operative CT imaging data were collected for all patients admitted with FNFs and treated with cannulated screws between February 2015 and August 2020. Inclusion criteria: (1) unilateral FNF; (2) fixed with cannulated screws; (3) aged 18 years and older. Exclusion criteria: (1) bilateral FNFs; (2) with concomitant proximal femoral fractures; (3) preoperative disability of the healthy or affected limb; (4) with combined malignant tumors and immune diseases; (5) pathological fractures.

## Measurement method

DICOM format CT images were imported into Mimics 22.0 (Materialize, Leuven, Belgium) for 3D reconstruction bilateral proximal femurs. The proximal femoral 3D models were reconstructed using a semi-automatic workflow. Initial segmentation was performed via region growing by placing desired and undesired seed points with a seed threshold of 175 HU. This was followed by precise filtering using global Hounsfield unit thresholds (typical range: lower limit 120–150 HU, upper limit 2000–3000 HU, adjusted as needed based on individual bone density). A gap closing distance of 8 pixels (px) was applied to refine the bone mask. 3D models were then created using the “Calculate 3D from Mask” command. All models underwent multi-angle visual inspection to ensure morphological integrity and exclusion of soft tissue. As the theoretical basis of this study, the human hip joint is symmetrical on both sides, which has been confirmed by multiple studies^27–29^. Using the mirroring function in Mimics software, a mirrored 3D model of the healthy side was generated. The 3D model of the fracture side was registered with the healthy side mirror through the registration function. The mirrored femur model was computationally superimposed onto the contralateral healthy femur, with alignment optimized through matching greater trochanter morphology and coaxial alignment of femoral shaft axes. After the reconstruction and registration of the healthy side’s mirrored femoral head and the affected side’s femoral head, both models were simultaneously selected and remeshed. The geometric centers of the two models were determined separately in the 3-matic software module. The position of the geometric center of the sphere is determined as center of femoral head. Another key point of the femoral head to be marked was the deepest point of fovea of femoral head. Three experienced observers independently completed the entire measurement workflow. Each observer followed a consistent yet independent protocol, which included medical image segmentation, 3D reconstruction of the femoral head, mirror alignment, model registration, remeshing, spherical center fitting, manual annotation of key anatomical landmarks and final calculation of displacement metrics. Throughout the process, the observers remained blinded to each other’s intermediate data or results, thereby ensuring complete independence of measurements. The measurement indicators are described in the following text.

## Measurement indicators

**Fig. 1:**
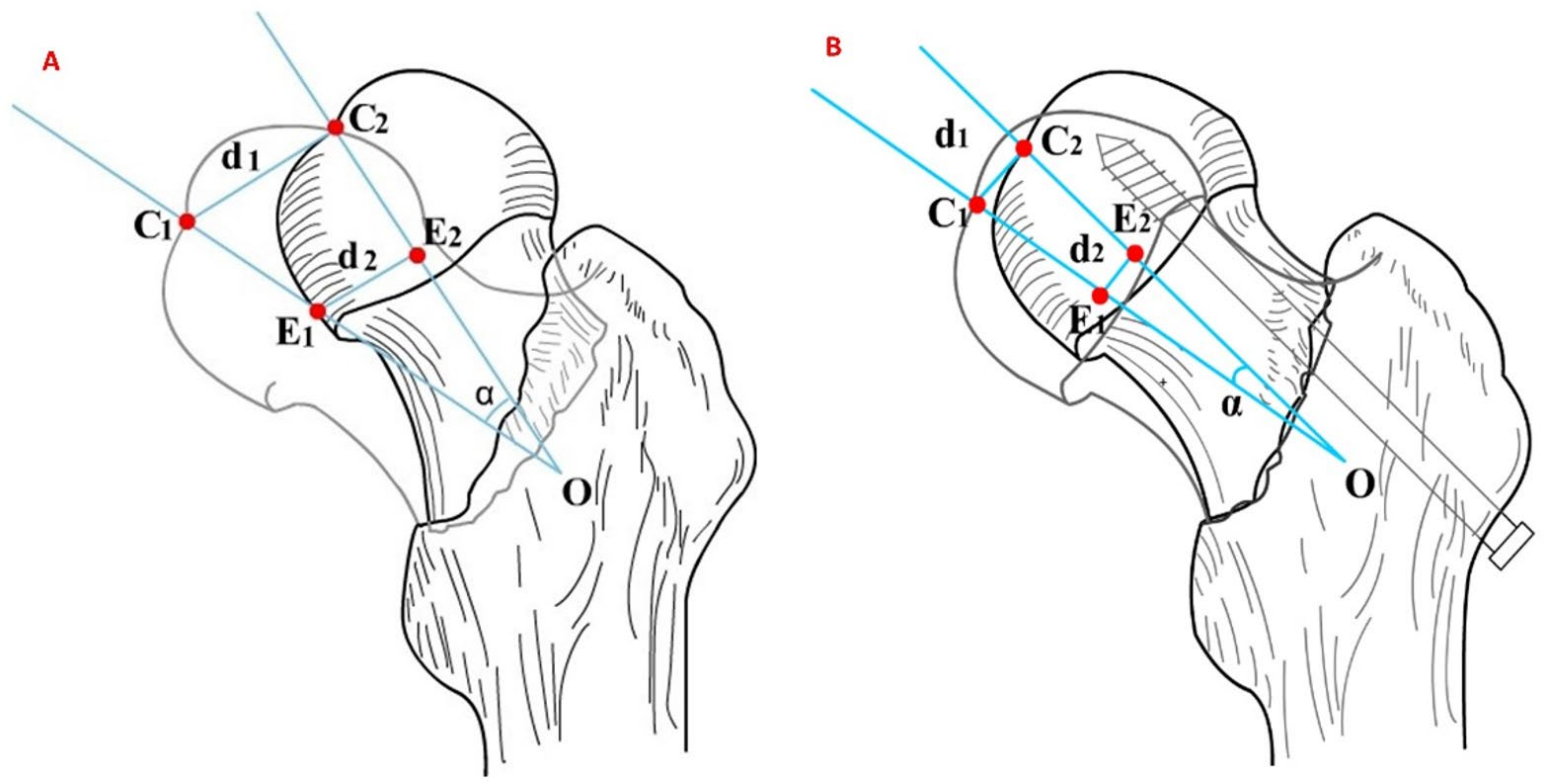
Schematic diagram of femoral head displacement before (**A**) and after (**B**) surgery in femoral neck fracture patients.

Displacement of fovea of femoral head (d1): The linear distance between the fovea of the femoral head of the affected-side (C1) and the mirrored contralateral side(C2). Displacement of center of femoral head(d2): The linear distance between the center of affected-side femoral head(E1) and the mirrored contralateral side(E2). 3D angle of femoral head (α): the angle between the line of affected-side fovea of the femoral head to center of the femoral head (C1E1) and the line of fovea of mirrored contralateral femoral head to center of the femoral head(C2E2). The schematic diagram of femoral head displacements in patients with FNFs before and after surgery is shown in Fig. 1A and B.

**Fig. 2:**
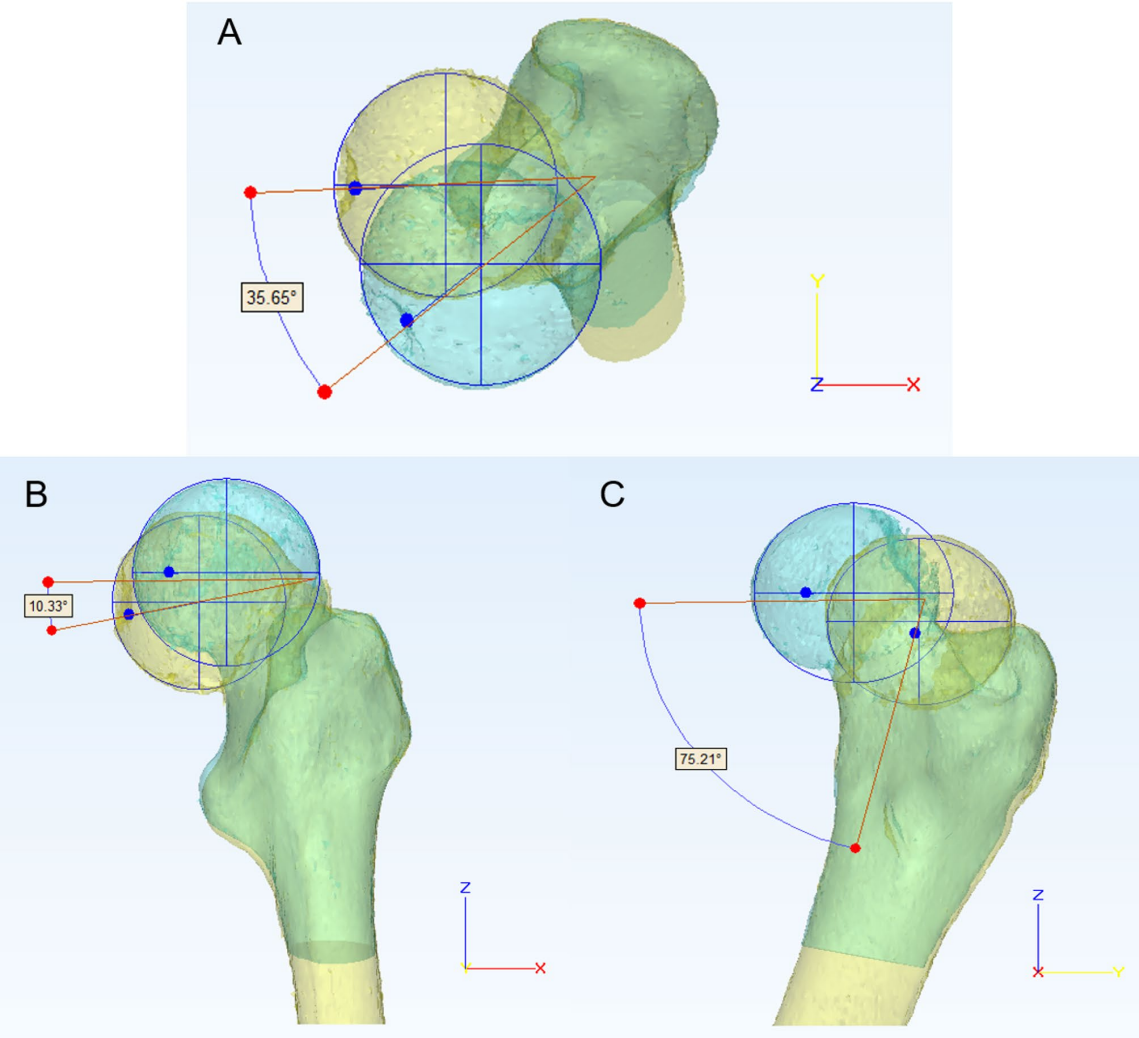
The projection angles of 3D spatial angles on three coordinate planes of a 43-year-old male patient before surgery, the projection angle on the transverse plane (X-Y) (**A**), projection angle on the coronal plane (X-Z) (**B**), and projection angle on the sagittal plane (Y-Z) (**C**).

The projection angles of 3D angle on three coordinate planes are also measured including projection angle on the transverse plane (X-Y), projection angle on the coronal plane (X-Z), and projection angle on the sagittal plane (Y-Z) (Fig. 2). The projection angle on the transverse plane (X-Y) clinically represents the degree of femoral head displacement—anterior (ventral)/posterior (dorsal) and left/right—compared to the original position, as shown in Fig. 2A; The projection angle on the coronal plane (X-Z) clinically quantifies the displacement of the femoral head fragment in the superior/inferior (head/foot) and left/right directions, which can be measured via anteroposterior (AP) radiographs or coronal CT reconstructions (Fig. 2B); The projection angle on the sagittal plane (Y-Z) represents the displacement of the femoral head fragment in the superior/inferior (head/foot) and anterior/posterior (ventral/dorsal) directions relative to its original position, measurable via lateral radiographs or CT reconstructions(Fig. 2C).

### Data analysis

All data were analyzed and graphically described using Python 3.10.0. Paired t-tests were used to compare preoperative and postoperative measurement indicators, where the analysis was performed on the data averaged across the three observers. The reliability of measurement indicators of 3D displacement of femoral head was calculated using ICC (Intraclass Correlation Coefficient) and CCC (Concordance Correlation Coefficient). ICC is the best-known reliability parameter for repeated measurements of continuous variables^30^. The derivation of the formula for ICC is based on the framework of analysis of variance (ANOVA). In this study, to evaluate the reliability and agreement among different observers, we employed the ICC for statistical analysis. Specifically, the average measure ICC based on a two-way random-effects, absolute agreement model was selected and reported as the primary metric to reflect the reliability and agreement across multiple observers. The formula is as follows:

$$\:ICC(A,k)=\frac{{\sigma\:}_{{s}^{2}}}{({\sigma\:}_{{s}^{2}}+\frac{{\sigma\:}_{{r}^{2}}}{k}+\frac{{\sigma\:}_{{e}^{2}}}{k})}$$

Where $\:A$ denotes the absolute agreement model, $\:k$ represents the number of observers; $\:{\sigma\:}_{{s}^{2}}$, $\:{\sigma\:}_{{r}^{2}}$ and $\:{\sigma\:}_{{e}^{2}}$ are the estimated variance components for subjects, observers, and residual error, respectively.

It should be noted, however, that the average measure ICC may overestimate the reliability and agreement of the method when used by a single observer. Therefore, to provide a more comprehensive assessment, the single measure ICC along with its 95% confidence interval is also presented in the Supplementary Material Table 1.

**Table 1:** Quantitative measurements of displacements and 3D angle of femoral head and Paired-Samples T Test.

Variables	Preoperative	Postoperative	T value	*P* value
3D angle of femoral head (°)	26.94 ± 12.12	12.84 ± 5.57	11.18	< 0.001
Displacement of center of femoral head(mm)	13.48 ± 7.41	5.51 ± 1.92	10.72	< 0.001
Displacement of fovea of femoral head(mm)	21.39 ± 11.59	8.37 ± 3.36	11.25	< 0.001
Projection angle on the transverse plane (X-Y) (°)	20.80 ± 13.19	9.16 ± 5.03	8.93	< 0.001
Projection angle on the coronal plane (X-Z)(°)	16.71 ± 11.57	8.34 ± 5.47	6.81	< 0.001
Projection angle on the sagittal plane (Y-Z) (°)	48.94 ± 35.15	22.35 ± 16.63	7.71	< 0.001

CCC combines Pearson correlation coefficient (measuring linear correlation) and bias correction factor (measuring consistency)^31^. For R observers, the formula is^32,33^:

$$\:{\rho\:}_{o}^{c}=\frac{2{\sum\:}_{r=1}^{R-1}{\sum\:}_{s=r+1}^{R}{\sigma\:}_{rs}}{\left(R-1\right){\sum\:}_{r=1}^{R}{{\sigma\:}_{r}}^{2}+{\sum\:}_{r=1}^{R-1}{\sum\:}_{s=r+1}^{R}{({\mu\:}_{r}-{\mu\:}_{s})}^{2}}$$

Here, $\:{\mu\:}_{r}$ and $\:{{\sigma\:}_{r}}^{2}$ denote the mean and variance for the *r*th observer, and $\:{\sigma\:}_{rs}$ is the covariance for observers *r* and *s*.

To further quantify the absolute range of measurement error and identify potential systematic bias, this study additionally applied the Standard Error of Measurement (SEM) and Bland-Altman Limits of Agreement (LoA) analysis. SEM reflects the dispersion of repeated measurements obtained by different observers using the same instrument when assessing identical subjects, representing the probable variation around the theoretical true value. The larger the SEM, the lower the test reliability. Bland-Altman LoA is defined as the range within which 95% of the differences between two measurement methods (or repeated measurements) are expected to fall.

The formula of SEM is:

$$\:SEM=\sigma\:\sqrt{(1-ICC)}$$

## Ethics

This study was approved by the Ethics Committee of Tianjin Hospital (approval no. 2015-049). All procedures were performed in accordance with the Declaration of Helsinki. Written informed consent was obtained from all participants.

## Results

A total of 200 CTs from 100 patients were included in this study (age: 63.5 ± 4.9 years; 56 female, 44 male). Displacement of center of femoral head, displacement of fovea of femoral head, 3D angle of femoral head and its projected angles onto the three planes of the coordinate axis changed significantly after internal fixation. The paired t-test results (Table 1) indicated that the postoperative measurement indicators are significantly lower than preoperative values, and the differences were statistically significant.

Inter-observer ICC coefficients for all measurements pre- and postoperatively are shown in Table 2. Inter-observer ICCs were great for all the preoperative measurements indicators. While for postoperative measurements indicators, inter-observer ICCs were good for displacement of center of femoral head and displacement of fovea of femoral head, and good to moderate for the rest. SEMs showed higher errors of measurement for angles on transverse plane (X-Y) (4.501° and 4.212°) and sagittal plane (Y-Z) (12.389° and 10.514°) both preoperatively and postoperatively.

**Table 2:** ICC coefficients and SEMs for all measurement indicators.

Variables	ICC	95%CI	*P* value	SEM(mm/°)
Preoperative 3D angle of femoral head (°)	0.925	0.896–0.948	< 0.001	3.565
Preoperative displacement of center of femoral head (mm)	0.977	0.968–0.984	< 0.001	1.145
Preoperative displacement of fovea of femoral head (mm)	0.982	0.974–0.987	< 0.001	1.622
Preoperative projection angle on the transverse plane (X-Y) (°)	0.903	0.865–0.932	< 0.001	4.501
Preoperative projection angle on the coronal plane (X-Z) (°)	0.918	0.886–0.942	< 0.001	3.564
Preoperative projection angle on the sagittal plane (Y-Z) (°)	0.894	0.852–0.926	< 0.001	12.389
Postoperative 3D angle of femoral head(°)	0.749	0.650–0.823	< 0.001	3.410
Postoperative displacement of center of femoral head(mm)	0.846	0.785–0.892	< 0.001	0.857
Postoperative displacement of fovea of femoral head(mm)	0.841	0.778–0.888	< 0.001	1.542
Postoperative projection angle on the transverse plane (X-Y) (°)	0.605	0.451–0.722	< 0.001	4.212
Postoperative projection angle on the coronal plane (X-Z) (°)	0.771	0.681–0.839	< 0.001	3.157
Postoperative projection angle on the sagittal plane (Y-Z) (°)	0.738	0.634–0.816	< 0.001	10.514

*ICC* Intraclass Correlation Coefficient;* SEMs* standard error of measurement;* 95% CI* 95% confidence interval.

Among the preoperative indicators, the overall CCCs were high, with coefficients ranging from 0.74 to 0.95; the pairwise CCCs among all three observers demonstrated good reliability and agreement, with coefficients ranging from 0.68 to 0.96. In the postoperative indicators, the overall CCCs among the three observers ranges from 0.34 to 0.64, and pairwise CCCs among the three observers ranged from 0.32 to 0.74. The CCCs for these three observers, were shown in Table 3.

**Table 3:** The overall and pairwise CCCs for all measurement indicators.

Variables	Overall CCC		Pairwise	Two raters’ CCC	
	Pre	Post		Pre	Post
3D angle of femoral head	0.804	0.496	Rater1 ~ 2	0.781	0.477
			Rater1 ~ 3	0.817	0.555
			Rater2 ~ 3	0.814	0.451
Displacement of center of femoral head	0.934	0.644	Rater1 ~ 2	0.923	0.652
			Rater1 ~ 3	0.925	0.628
			Rater2 ~ 3	0.954	0.653
Displacement of fovea of femoral head	0.946	0.635	Rater1 ~ 2	0.934	0.619
			Rater1 ~ 3	0.941	0.700
			Rater2 ~ 3	0.964	0.579
Projection angle on the transverse plane (X-Y)	0.754	0.336	Rater1 ~ 2	0.740	0.216
			Rater1 ~ 3	0.743	0.425
			Rater2 ~ 3	0.780	0.354
Projection angle on the coronal plane (X-Z)	0.787	0.526	Rater1 ~ 2	0.785	0.544
			Rater1 ~ 3	0.782	0.573
			Rater2 ~ 3	0.795	0.462
Projection angle on the sagittal plane (Y-Z)	0.737	0.481	Rater1 ~ 2	0.749	0.741
			Rater1 ~ 3	0.779	0.354
			Rater2 ~ 3	0.681	0.323

*CCC* Concordance Correlation Coefficient.

**Fig. 3:**
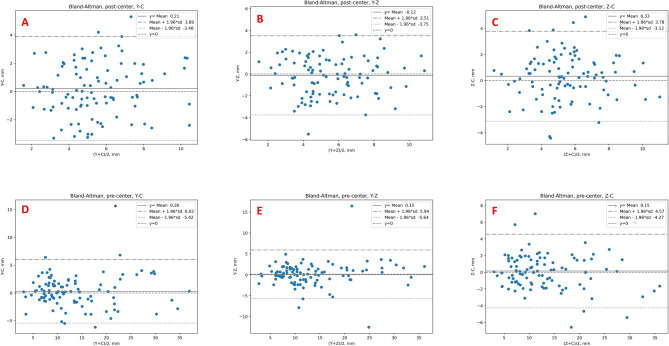
Bland-Altman analysis for measuring the displacement of center of femoral head. Bland-Altman analysis of pre- vs. postoperative measurements of displacement of center of femoral head among three observers (*n* = 100 patients). Solid black line represents the mean difference (bias), dashed black line indicates zero-difference reference, with dashed-dot and dotted black lines indicating 95% LoA (± 1.96 SD). Bland-Altman analysis of preoperative displacement of center of femoral head between observer_Y and observer_C (**A**), observer_Y and observer_Z (**B**), observer_Z and observer_C (**C**); Bland-Altman analysis of postoperative displacement of center of femoral head between observer_Y and observer_C (**D**), observer_Y and observer_Z (**E**), observer_Z and observer_C (**F**).

The Bland-Altman analysis demonstrated that all the pairwise mean differences between two observers were small, ranging from − 1 to 1(mm/°) for most pre and post measurement indicators, except for the sagittal plane (Y-Z) angle (−8.09 to 1.05) (Table 4). The Bland-Altman LoAs on every two observers for all the measurement indicators were calculated (Table 4), and the percentages of points within LoAs ranged from 92 to 97%, generally equal to the theoretical expectation of 95% (Table 4; Fig. 3, Supplementary Material Fig. 1, Supplementary Material Fig. 2, Supplementary Material Fig. 3, Supplementary Material Fig. 4, Supplementary Material Fig. 5). Take the post-displacement of center of femoral head for example, mean differences between two observers were − 0.12 mm (LoA: −3.75 to 3.51 mm), 0.21 mm (LoA: −3.48 to 3.89 mm), and 0.33 mm (LoA: −3.12 to 3.78 mm). The percentages of the points falling in the LoAs were 96%, 97%, and 92%(Fig. 3).

**Table 4:** Bland-Altman LoAs for all measurement indicators.

Variables	Pairwise	Mean differences		Bland-Altman LoAs	
		Pre	Post	Pre	Post
3D angle of femoral head (°)	Rater1 ~ 2	0.43	1.14	(-16.34 to 7.20)	(−11.58 to13.86)
	Rater1 ~ 3	−0.38	0.08	(−15.61 to 14.84)	(−13.11to 3.27)
	Rater2 ~ 3	−0.81	−1.06	(−16.29 to 14.66)	(−15.02 to 12.90)
Displacement of center of femoral head (mm)	Rater1 ~ 2	0.15	−0.12	(−5.64 to 5.94)	(−3.75 to 3.51)
	Rater1 ~ 3	0.30	0.21	(−5.42 to 6.02)	(−3.48 to 3.89)
	Rater2 ~ 3	0.15	0.33	(−4.27 to 4.57)	(−3.12 to 3.78)
Displacement of fovea of femoral head (mm)	Rater1 ~ 2	0.23	0.24	(−8.17 to 8.64)	(−6.16 to 6.63)
	Rater1 ~ 3	0.16	0.00	(−7.63 to 7.95)	(−6.07 to 6.08)
	Rater2 ~ 3	−0.07	−0.23	(−6.34 to 6.19)	(−7.04 to 6.58)
Transverse plane (X-Y) angle (°)	Rater1 ~ 2	−0.78	0.86	(−21.39 to 19.83)	(−15.00 to 16.73)
	Rater1 ~ 3	0.04	−0.67	(−19.85 to 19.92)	(−15.12 to 13.79)
	Rater2 ~ 3	0.81	−1.53	(−17.71 to 19.33)	(−16.33 to 13.27)
Coronal plane (X-Z) angle (°)	Rater1 ~ 2	0.20	0.75	(−10.12 to 10.52)	(−10.97 to 12.46)
	Rater1 ~ 3	−0.88	0.80	(−12.31 to 10.55)	(−11.43 to 13.04)
	Rater2 ~ 3	−1.08	0.06	(−11.89 to 9.73)	(−13.47 to 13.58)
Sagittal plane (Y-Z) angle (°)	Rater1 ~ 2	3.59	1.05	(48.80 to 55.98)	(−28.64 to 30.74)
	Rater1 ~ 3	4.50	−1.33	(−54.55 to 45.55)	(−46.60 to 43.94)
	Rater2 ~ 3	−8.09	−2.38	(−66.65 to 50.46)	(−47.67 to 42.92)

*LoAs* limits of agreement.

## Discussion

In this study, we systematically evaluated for the first time the reliability and agreement measurements of 3D displacements of femoral head in FNFs using MIMICS software’s 3D reconstruction technology among multiple observers. This study demonstrates that CT-based 3D reconstruction achieves an ICC indicating high reliability and agreement (ICC > 0.894) for preoperative displacement measurements. Furthermore, further analysis revealed that when anatomical reduction was achieved postoperatively, the ICC/CCC exhibited lower values. This phenomenon is likely attributable to their mathematical nature, which inherently relies on between-subjects variability. Bland-Altman analysis showed that ≥ 92% of individual measurements across all postoperative groups remained within the agreement limits, highlighting the necessity of adopting complementary metrics to construct a more comprehensive and robust evaluation framework. These findings provide critical methodological insights for micro-displacement quantification in fracture reduction assessment.

Current epidemiological data indicate that FNFs constitute approximately 3.6% of all fractures and represent 48–54% of hip fractures in the Chinese population^1^. Surgical intervention remains the gold-standard treatment for FNFs in medically fit patients. Internal fixation represents the first-line treatment for FNFs in patients aged < 65 years with satisfactory bone quality^34^. The goal of surgical treatment is to preserve the femoral head as much as possible, avoid femoral head necrosis, and achieve bone healing^35^. After internal fixation surgery for FNFs, the incidence of adverse complications such as non-union of the fracture and ischemic necrosis of the femoral head is relatively high. According to reports, the incidence of femoral head necrosis in young patients with FNFs is 14.3%, and the non-union rate of fractures is 9.3%^35,36^. A significant positive correlation exists among the initial displacement degree of FNFs, residual displacement after surgical reduction, and postoperative complication rates. Young patients with displaced fractures have a significantly higher incidence of complications compared to those without displaced fractures^36^.

Poor fracture reduction represents a significant high-risk factor, giving rise to various adverse outcomes. These include complications in fracture healing such as non—union, delayed union, and malunion, limited restoration of functionality, and the need for secondary revision surgeries^37–41^. In this study, we evaluated the degree of displacement of femoral head from three aspects, namely displacement of fovea of femoral head, displacement of center of femoral head and 3D angle of femoral head.

Accurately measuring relevant data is a necessary condition for conducting subsequent research^30^. MIMICS is widely used for measuring medical imaging data^42,43^. It can reconstruct the healthy and affected femoral heads in three dimensions and perform mirror processing to obtain the approximate anatomical position of the fractured femoral head, and measure the displacement and 3D angle of fractured femoral head. In our study, three researchers used MIMICS software to measure the same group of patients and conducted inter observer reliability and agreement analysis on the measurement data.

In this study, the ICC was used for reliability and agreement analysis^44^. The ICC range is between 0 and 1. Generally, a higher ICC value suggests better inter-observer reliability and agreement. It is often believed that ICC ≥ 0.75 indicates very good reliability and agreement, whereas values below 0.40 are considered poor^30,44,45^. In this study, the preoperative ICC range was between 0.894 and 0.982, and the postoperative ICC range was between 0.605 and 0.846. Incorporating metrics such as the Bland-Altman analysis, although the postoperative ICC values were lower than the preoperative levels due to narrowed data ranges, these results indicates that the three researchers have similar effects in measuring the displacement and 3D angle of femoral head in patients with FNFs using MIMICS software.

In order to further conduct reliability and agreement research, we also calculated the CCC. The preoperative CCC range is between 0.737 and 0.946, and the postoperative CCC range is between 0.336 and 0.635. Both indicators reflect a problem, that is, the ICC and CCC values of preoperative indicators are higher than postoperative indicators for the same group of patients and measurement personnel. Through literatures^30^ and formulas for ICC and CCC, it was determined that the reduction in postoperative ICC/CCC values, which primarily stems from the mathematical nature of these indices: both ICC and CCC inherently reflect the proportion of between-subjects variance relative to the total variance. Following surgical anatomical reduction, the between-subjects variability in postoperative measurements decreases substantially, thereby reducing this proportion. Consequently, the decrease in the coefficient does not necessarily indicate poorer measurement agreement, but rather may reflect a natural outcome of successful surgery- with a consequent reduction in inter-patient variability as the measurements cluster within a desired range. When between-subjects variability is low postoperatively, correlation coefficients can decrease even if the absolute measurement differences remain small. It should be noted in interpretation that lower ICC/CCC values in such contexts may “underestimate” the actual reliability and agreement of the measurement. Lin pointed out in proposing CCC that when the data range is narrow, the agreement estimation may be underestimated^31^. Barnhart studied multi-observer agreement and pointed out that when the data range is small, agreement indicators (such as CCC) may be lower^33^.

The Bland-Altman plot offers an effective way to visualize the variability between measurement values. The Bland Altman chart contains two important pieces of information: (1) the mean of the difference in measurements between two researchers, and the closer the mean is to 0, the better the agreement; (2) limit of agreement (LoA), which is the upper and lower 1.96SD of the average difference, the more points within this range, the better the agreement.

In order to further analyze the impact of different angles in different plane on the prognosis of FNFs and the ONFH, in this study, we also decomposed the 3D angle into angles on each plane. In the Bland Altman analysis, at least 92% of the points in the displacement and 3D angles of the fractured femoral head, as well as the angles in each plane, are within the LoA, and the LoA is relatively narrow. Bland-Altman analysis (Table 4) showed that the mean differences for most measurement indicators fell within the range of − 1 to 1 mm/°. However, a relatively large deviation was observed for the projection angle on the sagittal plane (–8.09 to 1.05). We conducted further analysis and propose that this is attributable to multiple interrelated factors: (1) During the registration process, the accurate localization of anatomical landmarks on the proximal femur model is challenging due to its deficient contour features in the sagittal plane. The researchers placed greater emphasis on matching the anatomical contours in the coronal plane (anteroposterior view) due to its more distinct features, while relatively neglecting the matching accuracy in the sagittal plane. This likely contributed to the larger errors observed in sagittal parameters; (2) The projection angle of a 3D angle onto the 2D sagittal plane is highly dependent on the selection of the projection plane. Unlike the coronal and transverse planes, where anatomical landmarks are relatively distinct, the visualization of the sagittal plane presents inherent challenges. Any minor deviation by the operator in positioning the software-defined “standard sagittal plane” inevitably affects the measurement accuracy of the final sagittal angle. Therefore, achieving precise and reproducible registration among the standard transverse, sagittal, and coronal planes remains a key issue to be addressed in future research; 3) Patients often cannot fully control pelvic rotation and lower limb positioning. During image registration, observers are influenced by the inherent angular variations between the bilateral femurs, leading to differing subjective judgments, which increases the difficulty of registration and ultimately contributes to measurement deviations. In summary, we posit that the deviations primarily originate from insufficient standardization in the methodology of 3D measurement. This limitation becomes particularly evident in the sagittal plane, which lacks distinct anatomical landmarks. In subsequent research, we aim to address this issue by achieving precise localization to standard anatomical planes and implementing semi-automated fitting algorithms for improvement. The primary objective of this study was to evaluate the reliability and agreement of 3D measurements performed by different researchers using Mimics software. Based on the overall results from various indicators presented in the manuscript, we consider this method generally reliable and applicable for future research. However, it is essential to clearly delineate the boundaries of its clinical application: The variability in sagittal projection angle revealed in this study suggests that indiscriminate use of these data in subsequent research—for instance, directly employing them to guide precise surgical planning or analyzing them as key factors influencing clinical outcomes—may affect the accurate assessment of therapeutic efficacy. Therefore, we recommend that sagittal plane-related parameters be interpreted with particular caution in future applications. This finding provides an important reference for standardizing the design of subsequent clinical studies and ensuring the correct interpretation of data. In this study, there was also a characteristic that the postoperative LoA range was smaller than before surgery, and the postoperative reduction effect of the severed end was significant.

This investigation demonstrated acceptable inter-observer reliability and agreement, supporting the use of data collected by different researchers in subsequent studies. However, a key limitation should be noted: standardized registration was not achieved across the three anatomical planes of interest, which contributed to increased errors in the 2D projection angles derived from the decomposition of 3D angles.

## Conclusion

In summary, while CT-based 3D reconstruction techniques have emerged as a valuable tool for assessing FNFs displacement and reduction quality, there remains a significant lack of standardized validation regarding the measurement agreement and reliability. This study demonstrates that CT-based 3D reconstruction achieves an ICC indicating high reliability (ICC > 0.894) for preoperative displacement measurements. However, postoperative assessments following reduction and fixation show a wider range of ICC values (moderate to good) and relatively lower CCCs. It should be noted that ICC/CCC values are influenced by inter-individual variability within the sample: when postoperative differences among patients are small, these coefficients may not fully reflect the actual measurement reliability/agreement. Notably, SEMs are higher for projection angles on transverse and sagittal planes, and Bland–Altman analysis reveals greater bias in the projection angle on the sagittal plane. It is important to acknowledge that advancements in future research—particularly through the precise definition and localization of the reference anatomical planes for femoral head 3D reconstruction, and the subsequent establishment of standardized registration protocols or development of semi-automated algorithms—would substantially improve the objectivity and reproducibility of the measurements, thereby enhancing the overall scientific rigor of the method.

## Supplementary Information

Below is the link to the electronic supplementary material.

Supplementary Material 1

Supplementary Material 2

Supplementary Material 3

Supplementary Material 4

Supplementary Material 5

Supplementary Material 6

## Data Availability

The data contain sensitive patient information and cannot be shared publicly to comply with institutional ethics committee requirements. Anonymized data may be available from the corresponding author on reasonable request.

## Abbreviations

FNFs: Femoral neck fractures
3D: Three-dimensional
d1: Displacement of fovea of femoral head
d2: Displacement of center of femoral head
α: 3D angle
ICC: Intraclass correlation coefficient
CCC: Concordance correlation coefficient
SEM: Standard error of measurement
LoA: Limits of agreement
