# Supplementary material for: Reliability and agreement study of three-dimensional measurement for femoral head displacement indicators after femoral neck fractures
Source: Sci Rep. 2026 Feb 27;16:11303. doi: 10.1038/s41598-026-41210-1 (PMC13049042; doi:10.1038/s41598-026-41210-1)
Supplement: Supplementary file 3 — Supplementary Material 3 [file 41598_2026_41210_MOESM3_ESM.pdf]

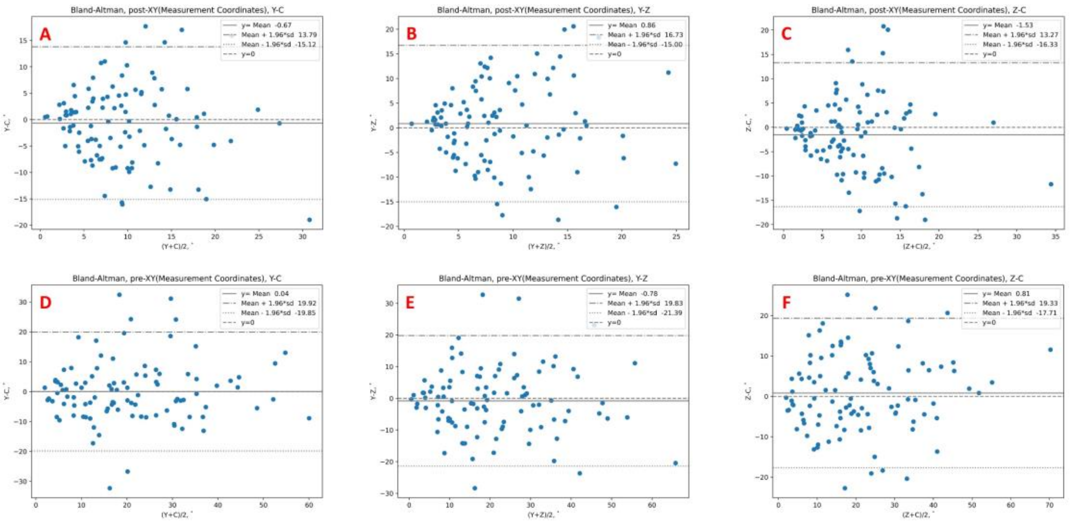

FigureS3. Bland-Altman analysis for measuring the projection angle on the transverse plane (X-Y). Bland-Altman analysis of pre- vs. postoperative measurements of projection angle on the transverse plane (X-Y) among three observers (n=100 patients). Solid black line represents the mean difference (bias), dashed black line indicates zero-difference reference, with dashed-dot and dotted black lines indicating 95% limits of agreement (±1.96 SD). Bland-Altman analysis of preoperative projection angle on the transverse plane (X-Y) between observer_Y and observer_C (A), observer_Y and observer_Z (B), observer_Z and observer_C (C); Bland-Altman analysis of postoperative projection angle on the transverse plane (X-Y) between observer_Y and observer_C (D), observer_Y and observer_Z (E), observer_Z and observer_C (F).
