# Supplementary material for: Reliability and agreement study of three-dimensional measurement for femoral head displacement indicators after femoral neck fractures
Source: Sci Rep. 2026 Feb 27;16:11303. doi: 10.1038/s41598-026-41210-1 (PMC13049042; doi:10.1038/s41598-026-41210-1)
Supplement: Supplementary file 6 — Supplementary Material 6 [file 41598_2026_41210_MOESM6_ESM.docx]

Supplementary Material Table 1. ICC_single_ coefficients for all measurement indicators

| Variables | ICC_single_ | 95%CI | *P* value |
| --- | --- | --- | --- |
| Preoperative 3D angle of femoral head (°) | 0.805 | 0.742-0.858 | <0.001 |
| Preoperative displacement of center of femoral head (mm) | 0.935 | 0.911-0.954 | <0.001 |
| Preoperative displacement of fovea of femoral head (mm) | 0.947 | 0.927-0.962 | <0.001 |
| Preoperative projection angle on the transverse plane (X-Y) (°) | 0.756 | 0.680-0.820 | <0.001 |
| Preoperative projection angle on the coronal plane (X-Z) (°) | 0.789 | 0.721-0.845 | <0.001 |
| Preoperative projection angle on the sagittal plane (Y-Z) (°) | 0.738 | 0.658-0.806 | <0.001 |
| Postoperative 3D angle of femoral head(°) | 0.499 | 0.383-0.609 | <0.001 |
| Postoperative displacement of center of femoral head(mm) | 0.647 | 0.549-0.733 | <0.001 |
| Postoperative displacement of fovea of femoral head(mm) | 0.638 | 0.538-0.726 | <0.001 |
| Postoperative projection angle on the transverse plane (X-Y) (°) | 0.338 | 0.215-0.464 | <0.001 |
| Postoperative projection angle on the coronal plane (X-Z) (°) | 0.529 | 0.416-0.635 | <0.001 |
| Postoperative projection angle on the sagittal plane (Y-Z) (°) | 0.484 | 0.366-0.596 | <0.001 |

ICC_single_: Intraclass Correlation Coefficient for single measure
